# Male Androgenetic Alopecia: Population-Based Study in 1,005 Subjects

**DOI:** 10.4103/0974-7753.58556

**Published:** 2009

**Authors:** DS Krupa Shankar, M Chakravarthi, Rachana Shilpakar

**Affiliations:** Department of Dermatology, Manipal Hospital, Bangalore, India

**Keywords:** Androgenetic alopecia, Hamilton Norwood classification, prevalence

## Abstract

**Context:**

Male androgenetic alopecia (AGA) is a common condition. There is limited information on its prevalence and patterns.

**Aims:**

(1). To find the prevalence and most common pattern (2). To correlate the age and pattern of alopecia.

**Settings and Design:**

Population-based study.

**Materials and Methods:**

This is a population-based study from the public. The selection was random. The method involved was asking the age and, if found to between 30 and 50 years, the scalp was examined for alopecia and the pattern was determined using the Hamilton Norwood classification.

**Results:**

Of 1,005 subjects, the youngest was 30 years old and the oldest 49 years old, with a mean age of 37.05 ± standard deviation 4.79. 39.2% of the subjects were in the age group of 30-35, 34.4% in the 36-40 year age group, 26.0% in the 41-45 years age group and 0.4% in the 46-50 years age group. Five hundred and eighty-three subjects (58%) had AGA, the most common type being grade II (27.27%) followed by grade I (22.12%) and grade III (21.78%). 47.5% (*P* = 0.003) had pattern alopecia in the 30-35 years age group, 58.7% in the 36-40 years age group (*P* = 0.8) and 73.2% in the 41-45 years age group (*P* ≤ 0.001). In the 30-35 years age group, grade I was 51.18%, grade II was 42.77% and grade VI was 18.52%. In the 41-45 years age group, grade I was 13.38%, grade III was 33.85% and grade VI was 66.67%.

**Conclusions:**

Fifty-eight percent of the male population aged 30-50 years had AGA. Its grade increased with increase in age. 12.9% of the male population had grades IV to VI, and would benefit from hair transplantation while 44.1% had grades I to III and are potential candidates for medical treatment

## INTRODUCTION

Hair loss is a common cosmetically and psychosocially distressing condition. Androgenetic alopecia (AGA) is the most common cause of hair loss among males.[1] Although the age of onset has been documented at 40 years, there are evidences that show that alopecia begins at 30 years of age.[2] But, this condition attracted least attention and there are limited studies on its prevalence and its grade in the Indian subcontinent. It is important to have further knowledge regarding its prevalence, grade of alopecia and its natural course for providing the appropriate management.

## MATERIALS AND METHODS

This is a population-based study from the public within the vicinity of our hospital, involving 1,005 men between 30 and 50 years of age who were willing to be a part of the study. The period of study was 1 week. The selection was random and did not involve dermatology patients. We randomly selected the males who entered our hospital, which included patients, attainders, doctors and other hospital staff.

The age of the person was enquired. If it was found to be between 30 and 50 years, the scalp was examined with his oral consent for AGA and the pattern was determined using the Hamilton and Norwood classification. Photographs have been taken only from our hospital employees with their informed consent.

## RESULTS

A total of 1,005 men were included in the study, of which the youngest was 30 years of age and the oldest was 49 years old, with a mean age of 37.05 ± standard deviation 4.79. 39.2% of the subjects were in the age group of 30-35 years, 34.4% in the age group of 36-40 years, 26.0% in the 41-45 years age group and 0.4% in the 46-50 years age group [Table 1].

**Table 1:** Age distribution

Age in years	Number	%
30-35	394	39.2
36-40	346	34.4
41-45	261	26.0
46-50	4	0.4
Total	1,005	100.0
Mean ± SD	37.05 ± 4.79	-

**Figure 1:**
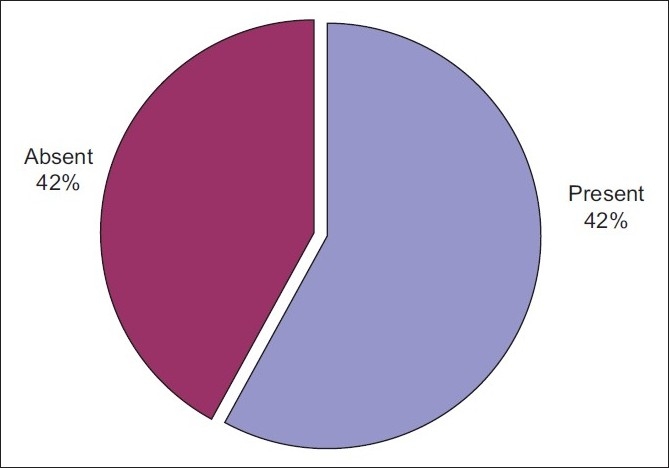
Prevalence of baldness

**Figure 2:**
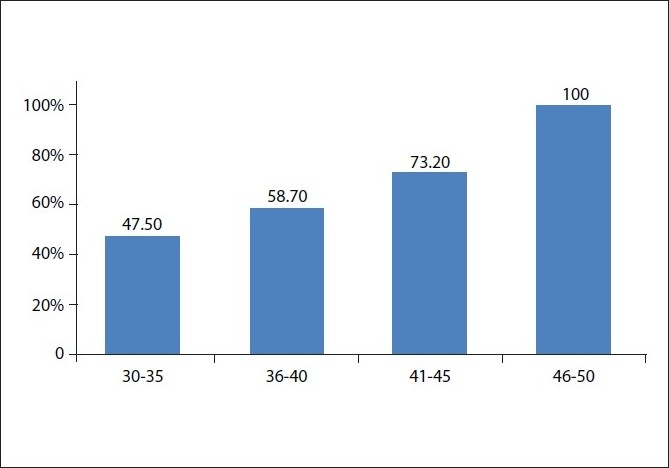
Percentage of androgenetic alopecia in each age group

Five hundred and eighty-three subjects (58%) had AGA while 422 subjects (42%) did not have alopecia [Figure 1]. The prevalence of AGA was 47.5% (*P* = 0.003) in the 30-35 years age group, 58.7% (*P* = 0.8) in the 36-40 years age group and 73.2% (*P* ≤ 0.001) in 41-45 years age group. Thus, the prevalence of AGA was statistically strongly significant and directly proportional to the increase in age [Table 2, Figure 2].

**Table 2:** Association of age with baldness

Age in years	Total number	Baldness		
		No	%	*P*-value
30-35	394	187	47.5	0.003**
36-40	346	203	58.7	0.862
41-45	261	191	73.2	<0.001**
46-50	4	4	100.0	0.089+
Total	1,005	583	58.1	‐

**Figure 3:**
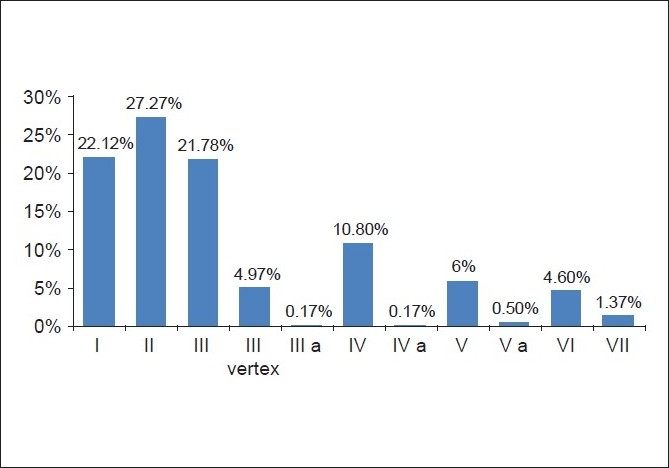
Percentage of each grade

The most common pattern of alopecia was grade II (27.27%), followed by grade I (22.12%) and then grade III (21.78%) [Figure 3].

The grade of alopecia was noted to increase with increase in age, as in the 30-35 years age group, grade I was 51.18%, grade II was 42.77% and grade VI was 18.52% while in the 41-45 years age group, grade I was 13.38%, grade III was 33.85% and grade VI was 66.67%.

## DISCUSSION

AGA is the most common type of hair loss, representing quantitative phenotype with an underlying genetic disposition along with androgen influence.[3] Pre-programmed follicles on the scalp undergo a transformation from long growth (anagen) and short rest (telogen) cycles to long rest and short growth cycles coupled with progressive miniaturization of the follicle.[3,4] These changes are androgen dependent and require the inheritance of several genes. The gene that encodes the androgen receptor has been identified.[4] Recently, the EDA2R gene that encodes the androgen receptor has been proposed to be associated with AGA.[5]

The evolution of baldness progresses from thinning in the temporal areas producing a reshaping of the anterior part of the hairline (temporal recession) then on to the loss of hair from the vertex region.[2] The grade of male AGA can be assessed using Hamilton[6] and Norwood's[7] classification.

In our study, we conclude that the prevalence of AGA in the male population between the ages of 30 and 50 years is 58%. A similar result has been quoted by Ellis *et al.,*[4] Thomas,[2] Bader *et al*.[8] and Otberg *et al.,*[9] who concluded that prevalence of AGA is approximately 50% in the population.

The prevalence increases with increase in age. The grade of alopecia gradually increases from the earlier grades (I and II) to the more severe type as the age increases. Grade II is the most common type of AGA, accounting to 27.27%.

Minoxidil and fenasteride are known to be effective medical treatments in AGA, especially during the initial grades.[9] Hair transplantation is useful in the moderate to severe grade of AGA. 12.9% of the male population has grade IV to VI, and would benefit from hair transplantation, while 44.1% had grades I to III and are potential candidates for medical treatment.
